# Generation of a glycogen storage disease mouse model with fatty liver and its treatment by PPAR-α agonist bezafibrate

**DOI:** 10.3389/fphar.2026.1898810

**Published:** 2026-08-31

**Authors:** Yixia Xie, Guoquan Fu, Caixia Fan, Huarong Huang, Junyan Yan, Lifang Jin

**Affiliations:** 1 School of Life and Environmental Sciences, Shaoxing University, Shaoxing, Zhejiang, China; 2 Hangzhou Hongwang Medical Laboratory Co. Ltd, Hangzhou, Zhejiang, China; 3 College of Life and Environmental Science, Hangzhou Normal University, Hangzhou, China

**Keywords:** fatty liver, glucose-6-phosphatase catalytic (G6PC), glycogen storage disease type ia (GSDIa), mouse model, ppar-α agonist

## Abstract

**Background:**

Fatty liver is considered the pathological basis for the development of glycogen storage disease type Ia (GSDIa); however, due to the lack of suitable animal models, the mechanism underlying fatty liver development in GSDIa is largely unknown.

**Methods:**

This study employed surrogate breastfeeding to enable the survival of glucose-6-phosphatase catalytic (*G6PC*) knockout (KO) mice into adulthood, and then analyzed fatty liver phenotypes in 3-month-old KO (KO-3), 6-month-old KO (KO-6), and 9-month-old KO (KO-9) mice.

**Results:**

KO-6 and KO-9 mice exhibited typical fatty liver phenotypes, and upregulation of glucose-6-phosphate (G6P). Moreover, both Western blot and qRT-PCR analysis showed significant upregulation of ChREBP, SREBP1 and fatty acid synthesis enzymes. RNA-seq heat maps revealed that KO-6 and KO-9 mice exhibited similar expression patterns, distinct from those observed in the wild-type (WT) and KO-3 groups. GO and KEGG analysis further revealed changes in lipid metabolism pathways in KO-6 and KO-9 mice, as well as PI3K-Akt, AMPK, and MAPK signaling. Western blot analysis showed that the Akt and mTOR pathways were activated, while the AMPK pathway was downregulated, indicating impaired autophagy. Finally, bezafibrate, a proliferator-activated receptor α (PPAR-α) agonist, enhanced autophagic activity and attenuated inflammatory responses, fat deposition, and glycogen storage in *G6PC* KO mice.

**Conclusion:**

This study successfully generated a fatty liver model using *G6PC* KO adult mice and demonstrated that pharmacological activation of PPAR-α may have therapeutic potential in adult *G6PC* KO mice.

## 1 Introduction

Glycogen storage disease type Ia (GSDIa) is a genetic disease affecting glucose metabolism, characterized by fasting hypoglycemia, hepatomegaly, hyperlipidemia, hyperuricemia, lactic acidemia, and growth retardation (Zhang et al., 2020). The primary cause of GSDIa is a deficiency in glucose-6-phosphatase catalytic subunit (G6PC), an enzyme primarily expressed in the liver and kidney, which can hydrolyze glucose-6-phosphate (G6P) into glucose during gluconeogenesis and glycogenolysis (Mutel et al., 2011). Consequently, *G6PC* deficiency can result in G6P deposition and glycogen storage, triggering the synthesis of fatty acid and severe nonalcoholic fatty liver disease. In a previous retrospective survey, Zhang et al. found that 98.6% of GSDIa patients presented with hypertriglyceridemia, while another 58.3% had hypercholesterolemia (Zhang et al., 2020). Wang et al. also reported a significant increase in the incidence of hepatic adenomas in GSDIa patients when triglyceride (TG) concentrations exceeded 5.6 mmol/L (Wang et al., 2011). Furthermore, in an observational study of 53 patients, high serum TG concentrations in childhood were associated with the development of hepatocellular adenomas in GSDIa patients (Haring et al., 2022).

Animal models, including mice and dogs, with complete *G6PC* deficiency or knockout (KO), have been utilized to mimic the characteristic features of human GSDIa disease, such as hyperlipidemia and fatty liver (Crane et al., 2012; Gautam et al., 2013). However, the survival of untreated KO pups beyond the weaning stage is rare, which limits the assessment of long-term liver pathogenesis. Salganik et al. employed glucose injection and standard chow administration to allow global *G6PC* KO mice survival into adulthood, and authors observed liver histology disorders and glycogen accumulation, but not fatty liver in adult *G6PC* KO mice (Salganik et al., 2009). Conversely, other studies have demonstrated that specific KO of hepatic *G6PC* can lead to the accumulation of hepatic G6P, glycogen, and TG, resulting in the development of fatty liver (Mutel et al., 2011; Peng et al., 2009). Therefore, the generation of a fatty liver model from adult mice with global *G6PC* KO has not yet been achieved.

The hepatic accumulation of G6P stimulates *de novo* lipogenesis via the glycolytic pathway (Chiu et al., 2018) and carbohydrate-responsive element-binding protein (ChREBP)-mediated lipid biosynthesis (Lei et al., 2020; Régnier et al., 2023). GSDIa patients exhibit reduced and impaired lipid clearance from the blood compartment (Bandsma and Kuipers, 2002; Hoogerland et al., 2021). Recent studies have also revealed the crucial role of autophagy in the development of fatty liver in *G6PC* KO mice. For example, Farah et al. demonstrated impaired autophagic activity during fatty liver development in neonatal *G6PC* KO mice, attributed to the downregulation of the AMPK pathway and upregulation of the mTOR pathway (Farah et al., 2016). Additionally, Gautam et al. discovered that upregulation in AMPK signaling increased autophagic activity in neonatal *G6PC* KO mice, thereby promoting lipolysis and attenuating fatty liver development (Gautam et al., 2020). Nonetheless, the precise mechanism underlying autophagy impairment in GSDIa-associated fatty liver remains elusive.

Bezafibrate is a clinically used lipid-lowering drug and a pharmacological agonist of PPAR-α, a nuclear receptor that plays a central role in hepatic fatty acid β-oxidation (Nakajima et al., 2010). Since impaired lipid catabolism contributes to hepatic steatosis in GSDIa, activation of PPAR-α may represent a rational therapeutic strategy. In addition, emerging studies suggest that PPAR-α signaling is linked to autophagy regulation under metabolic stress, further supporting investigation of bezafibrate in this model (Waskowicz et al., 2019).

In the current study, we successfully established a fatty liver model using global *G6PC* KO adult mice. Based on the model, we observed impaired autophagic activity during fatty liver development. Furthermore, our findings revealed that administration of bezafibrate, a proliferator-activated receptor α (PPAR-α) agonist, exerted regulatory effects on autophagic activity and fatty liver development in adult *G6PC* KO mice. Hence, our study contributes to a better understanding of GSDIa and provided potential treatment strategies for clinical interventions.

## 2 Materials and methods

### 2.1 Mice

The *G6PC* KO mice used in this study were generated previously by CRISPR-Cas9-mediated genome editing as described in our earlier report (Xie et al., 2022). Briefly, two sgRNAs targeting the intronic regions flanking Exon 3 of the murine *G6PC* gene were designed to induce Exon 3 deletion. Detailed sgRNA sequences, genomic coordinates, and validation of the model have been reported previously. After weaning and genotyping, pups were separated by sex and housed in different cages. Only male mice were used in all subsequent experiments. Sample sizes were determined based on prior experience with this model, expected effect sizes, and practical feasibility given the relatively low survival rate of global *G6PC* KO mice. To ensure adequate group sizes, model generation and animal inclusion were conducted in multiple rounds. Only animals that survived to the predefined experimental time point and met the health criteria for analysis were included (n = 7 per group). *G6PC* KO mice and wild-type (WT) mice were separately housed in plastic cages with a sawdust-covered floor under a constant temperature (26 °C) and 12:12-h light-dark cycle with free access to standard laboratory chow and tap water. Following blood collection, the mice were euthanized by cervical dislocation under isoflurane anesthesia, and liver tissues were collected and placed on ice for subsequent experiments. All animals received human care and the study protocols complied with the institution’s guidelines. During surrogate breastfeeding, pups were monitored at least once daily for survival, general appearance, spontaneous activity, nursing status, hydration, and body condition. Special attention was paid to milk intake, as indicated by the presence of a visible milk spot, as well as body weight gain and signs of poor maternal care or distress. Humane endpoints were predefined in accordance with institutional animal welfare guidelines. Pups showing persistent failure to nurse, severe weakness, marked weight loss, dehydration, or moribund condition were removed from the study and managed appropriately.

### 2.2 Intervention with PPAR-α agonist bezafibrate

The WT and *G6PC* KO mice (6 months old) were divided into three groups for treatment (WT + phosphate-buffered saline (PBS), KO-6 + PBS, and KO-6 + Bezafibrate). The *G6PC* KO mice (KO-6 + Bezafibrate) were gavaged daily with 75 mg/kg of bezafibrate (MCE, New Jersey, USA) for 1 month. As a control, WT mice (WT + PBS) and *G6PC* KO mice (KO-6+PBS) were treated with an equivalent volume of PBS.

### 2.3 Histology

The liver tissues were embedded in paraffin and cut into 4–5-µm sections. Hematoxylin and eosin (H&E) staining was according to standard protocols. Periodic acid Schiff (PAS) staining of the liver was performed using a PAS Staining Reagents System (Solarbio, Beijing, China) according to the manufacturer’s instructions. Liver tissue sections were immunostained with primary antibodies against myeloperoxidase (MPO, CST, Boston, USA), a critical enzyme in antimicrobial host-defense, and F4/80 (CST, Boston, USA), a surface marker of murine macrophages. Immunostained sections were then stained with horseradish peroxidase (HRP)-conjugated secondary antibodies and counterstained with hematoxylin. Hepatic fat accumulation was assessed by frozen sectioning and Oil Red O staining, with lipid droplets dyed red per the instructions of the Oil Red O staining kit (Solarbio, Beijing, China). For quantitative analysis, five randomly chosen visual fields were examined using a microscope, followed by ImageJ analysis.

### 2.4 Metabolite determination

Blood samples were centrifuged at 5 000 rpm for 5 min to obtain serum samples. Serum levels of free fatty acids (FFA), triglyceride (TG), and cholesterol (CHO) were measured using corresponding detection kits (Delta, Zhejiang, China). Hepatic G6P was measured with a G6P assay kit (Beyotime, Shanghai, China) according to the manufacturer’s instructions.

### 2.5 Quantitative real-time polymerase chain reaction (qRT-PCR)

Total RNA was extracted from liver tissues with Trizol reagent, and complementary DNA (cDNA) was synthesized with a cDNA reverse transcription kit (Takara, Osaka, Japan). PCR amplification was performed using a LightCycler 480 II instrument (Roche, Switzerland), along with SYBR Green Supermix, cDNA, and specific primers (listed in Supplementary Table S1). Relative gene expression changes were calculated using the 2^−ΔΔCT^ method.

### 2.6 Western blot analysis

Tissues were homogenized in RIPA lysis buffer (Solarbio, Beijing, China) to extract total protein. The primary antibodies used for Western blot analysis included p-Akt (Ser473, CST, Boston, USA), Akt (CST, Boston, USA), p-mTOR (Ser2448, CST, Boston, USA), mTOR (CST, Boston, USA), AMPK alpha 1 (phospho Thr183, CST, Boston, USA), AMPK alpha 2 (phospho Thr172, CST, Boston, USA), AMPK(CST, Boston, USA), ChREBP (Novus, Colorado, USA), SREBP (Novus, Colorado, USA), G6Pase-β (Origene, Maryland, USA), and β-actin (Hubio, Shanghai, China). Western blot band intensities were quantified by densitometric analysis using ImageJ software and normalized to the corresponding loading control. Quantification was performed using independent biological replicates.

### 2.7 Statistical analysis

Statistical comparisons between two groups were performed using a two-tailed Student’s t*-*test; multiple groups were analyzed by one-way analysis of variance (ANOVA) (GraphPad Prism v9.0). Statistically significant differences were determined at *P* < 0.05. The differentially expressed gene (DEG) screening criteria were |log2Fold-change| > 1 and adjusted *P*-value <0.05.

## 3 Results

### 3.1 Surrogate breastfeeding improved weaning survival in *G6PC* KO mice

In our previous study, we used CRISPR-Cas9 gene editing to generate a global *G6PC* KO mouse model (Xie et al., 2022). In the present study, to ensure the accuracy of the experiment, we conducted gene identification analysis of offspring mice derived from the model. To confirm the KO of the third exon of total *G6PC*, we analyzed five potential off-target sites for the Cas9 protein mediated by sgRNA1 and sgRNA2 (Supplementary Table S2). As shown in Supplementary Figures S1. 2, no large fragment insertions, deletions, or mutations were detected at the 10 potential off-target sites, suggesting that these global *G6PC* KO mice can be used for subsequent experiments.

**FIGURE 1:**
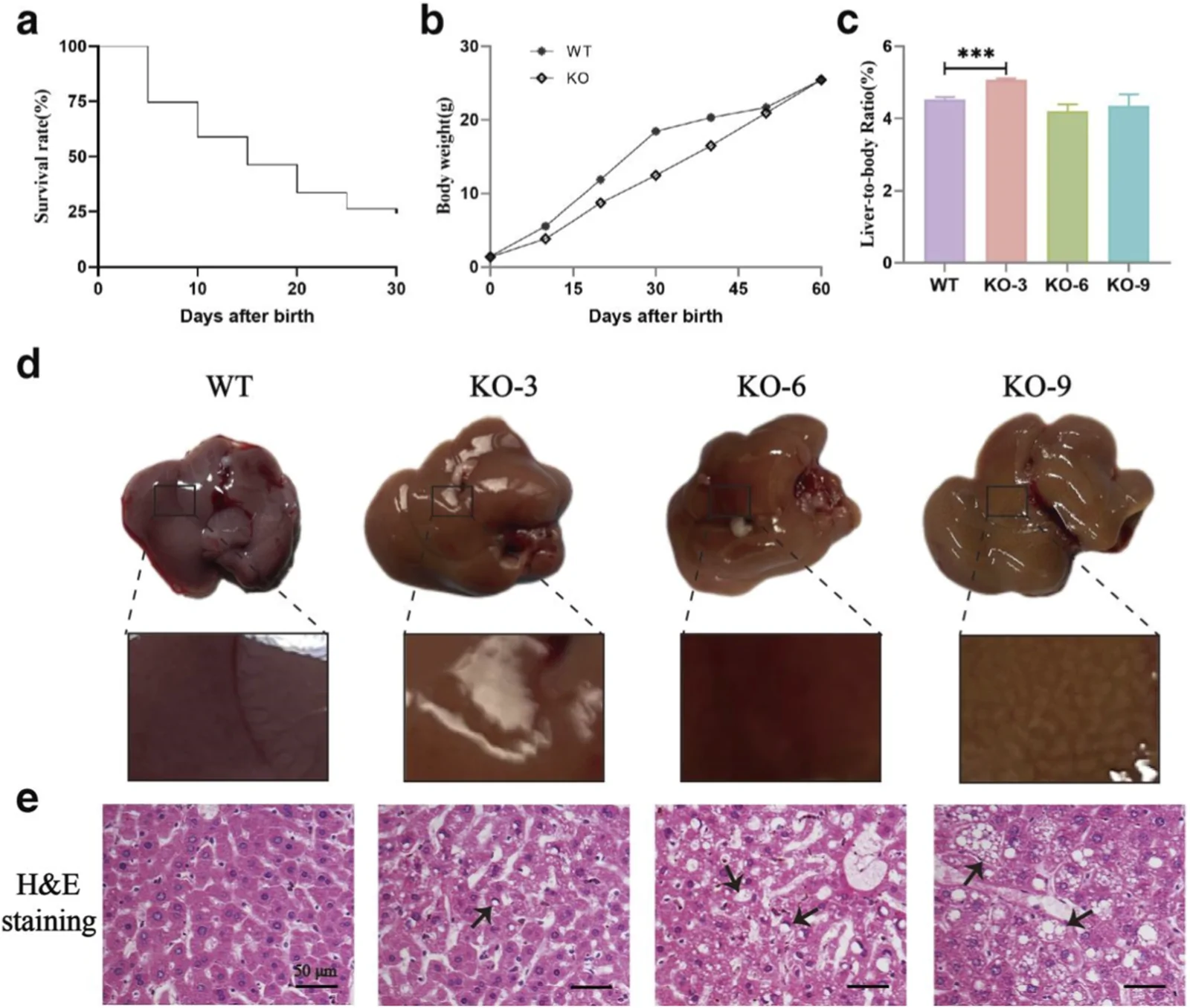
Changes in *G6PC* KO pups after surrogate breastfeeding care. **(a)** Survival rate of *G6PC* KO mice at 1 month. **(b)** Body weight of *G6PC* KO mice at 2 months. **(c)** Liver-to-body weight ratios in 3-, 6-, and 9-month-old *G6PC* KO adult mice. **(d)** Macroscopic appearance of liver in WT and 3-, 6-, and 9-month-old *G6PC* KO adult mice. **(e)** H&E-stained sections of liver from WT and 3-, 6-, and 9-month-old *G6PC* KO adult mice. Scale bar = 50 μm. Significantly different values from the WT group are indicated (***P < 0.001).

As described in previous research (Gautam et al., 2013; Xie et al., 2022), mice that were homozygous null for *G6PC* present with low birth weight, quickly develop severe and unremitting hypoglycemia, and seldom survived weaning. To improve the survival of *G6PC* KO mice during the weaning period, we used a surrogate breastfeeding approach to ensure that the *G6PC* KO mice received sufficient milk and care. Typically, one nursing mother mouse was assigned to feed one to two *G6PC* KO mice. Under these conditions, 24.2% (23/95) of *G6PC* KO mice survived up to 1 month. The survival rate of pups at different ages is shown in Figure 1a. Body weight measurements revealed that 30-day-old *G6PC* KO pups weighed approximately one-third less than their WT counterparts, while there was no significant difference between the KO and WT groups beyond 2 months of age (Figure 1a). Consistent with the finding of Salganik et al., *G6PC* KO mice that survived after weaning exhibited normal life activities (Salganik et al., 2009). However, after 2 months, the *G6PC* KO mice exhibited marked improvements in mobility and activity. In this study, we analyzed the liver-to-body ratios of adult WT and *G6PC* KO mice at 3 (KO-3), 6 (KO-6), and 9 months of age (KO-9). Results showed that the KO-3 group displayed a significant increase in the liver-to-body ratio compared to the WT group. However, this observed trend was not evident in the KO-6 and KO-9 groups (Figure 1c).

Based on liver morphology examination, the livers in the WT and KO-3 groups were bright red with no white plaques present. In contrast, the livers in the KO-6 and KO-9 groups were lighter in color and contained some white plaques, indicating pathological changes in the liver, such as fat accumulation (Figure 1d). To assess potential damage to the liver in *G6PC* KO mice, we analyzed liver histology. H&E staining showed that the KO-6 and KO-9 mice exhibited disrupted hepatic tissue and hepatocyte necrosis. Of note, we also observed many lipid vacuoles within hepatocytes from the KO-6 and KO-9 mice, further indicating the development of fatty liver (Figure 1e).

### 3.2 Inflammatory response, fat deposition, and glycogen storage in global *G6PC* KO adult mice

**FIGURE 2:**
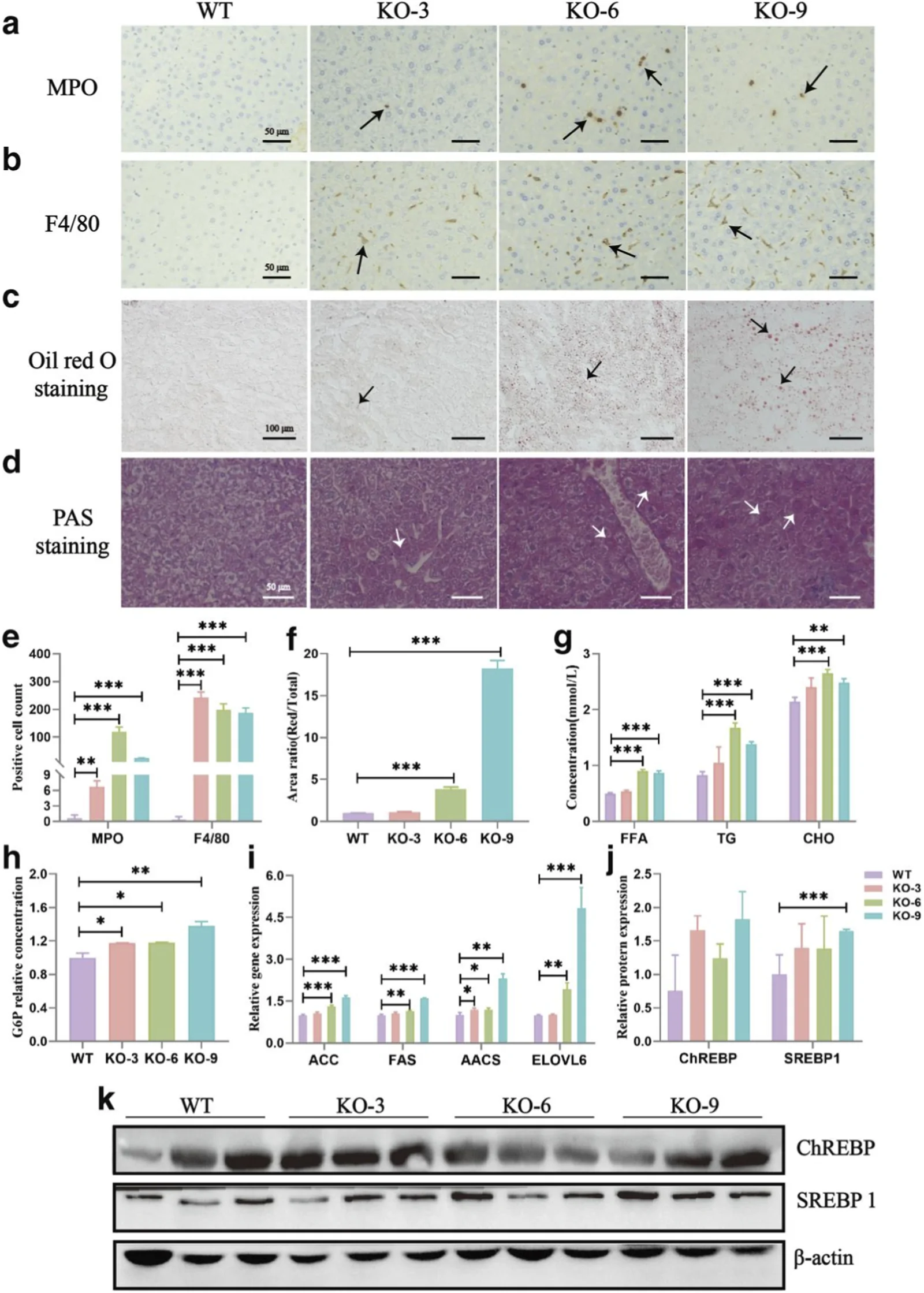
Characterization of *G6PC* KO adult mice. **(a,b)** Immunohistochemical staining of MPO and F4/80 in livers. Scale bar = 50 μm. **(c)** Representative liver tissue images of Oil Red O staining and **(f)** quantitative analysis of Oil Red O staining. Scale bar = 100 μm. **(d)** PAS-positive stained material. Scale bar = 50 μm. **(e)** Quantification of positive cell numbers in panels **(a,b)**. **(g)** Concentrations of free fatty acids (FFA), triglycerides (TG), and cholesterol (CHO) from serum panels in *G6PC* KO mice. **(h)** Relative hepatic G6P levels. **(i)** Relative expression level of *ACC*, *FAS*, *AACS*, and *ELOVL6* genes. **(j)** Relative protein expression levels of ChREBP and SREBP 1 in liver. **(k)** Representative Western blot images of ChREBP and SREBP 1 in liver. n = 6. Significantly different values from WT group (*P < 0.05; **P < 0.01; ***P < 0.001) are indicated. Quantitative analysis was performed using independent biological replicates (n = 3) and normalized to β-actin.

We also observed a significant increase in the number of MPO-positive neutrophils and F4/80-positive macrophages in the KO groups, indicating an inflammatory response in *G6PC* KO mice (Figures 2a,b,e). Considering the presence of lipid vacuoles in hepatocytes from KO-6 and KO-9 mice, we analyzed fatty liver development in adult *G6PC* KO mice of different ages. The results revealed evident white fat deposition in the livers of KO-6 and KO-9 mice. Oil Red O staining further demonstrated that the livers of KO-6 and KO-9 mice had more pronounced fatty deposition compared to those of WT and KO-3 mice (Figures 2c,f). Moreover, the intensity of PAS staining, which strongly reflects glycogen particle content, displayed a strong red-purple color in the cytoplasm of hepatocytes. Notably, in the KO-6 and KO-9 groups, intense PAS staining was observed in the cytoplasm of all hepatocytes, indicating the accumulation of glycogen (Figure 2d).

We next detected the concentration of serum lipid markers in the blood of WT and KO mice. Results showed that the concentrations of FFA, TG, and CHO were significantly higher in KO-6 and KO-9 mice than in WT and KO-3 mice (Figure 2g). Deficiency of *G6PC* results in G6P accumulation due to impairment of the gluconeogenesis and glycogenolysis pathways. Here, we detected the concentration of G6P in the livers of *G6PC* KO mice. As expected, the concentration of G6P was significantly increased in adult *G6PC* KO mice, especially in 6- and 9-month-old mice (Figure 2h). Collectively, these findings provide compelling evidence for the development of fatty liver in adult *G6PC* KO mice.

To quantify the expression levels of fat synthesis-related enzymes, qRT-PCR was conducted. As shown in Figure 2i, the gene expression levels of *acetyl-CoA carboxylases (ACC)*, *fatty acid synthase (FAS)*, *acetoacetyl-CoA synthetase (AACS)* and *elongase of very long chain fatty acids 6 (ELOVL6)* were significantly elevated in the *G6PC* KO mice. Consistent with the fatty liver phenotype, the activity of transcriptional regulators modulating lipogenesis was increased in the *G6PC* KO mice, especially in the KO-9 group. ChREBP and SREBP1 are major transcriptional regulators known to control lipogenesis in the liver (Crewe et al., 2019). Thus, we detected the expression levels of hepatic ChREBP and SREBP1 in WT and *G6PC* KO mice. Western blot analysis showed that the expression levels of ChREBP and SREBP1 were increased in the *G6PC* KO mice compared to the WT mice (Figures 2j,k).

### 3.3 RNA-seq analysis of liver transcriptome in *G6PC* KO mice

**FIGURE 3:**
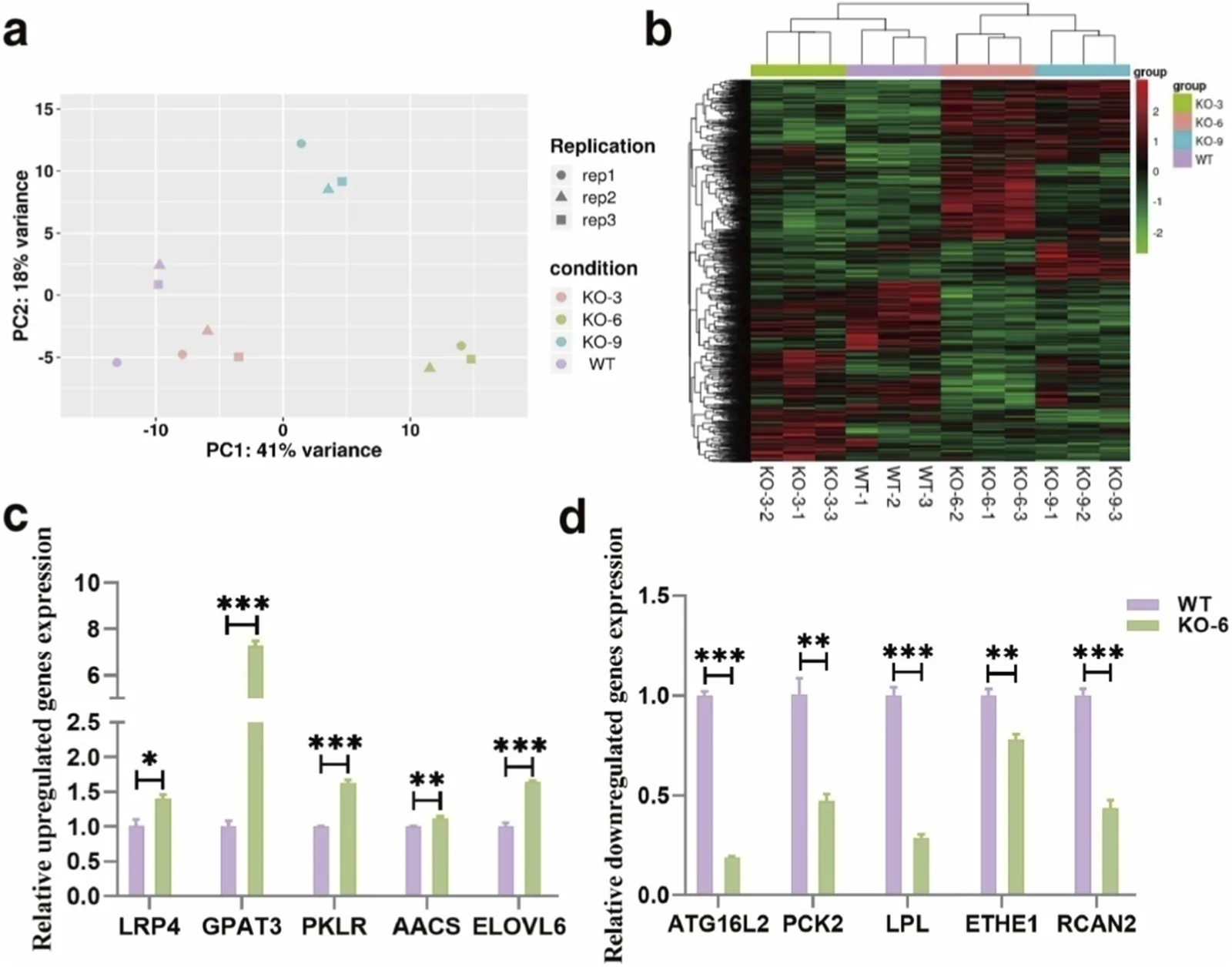
RNA-seq analysis of liver transcriptome in *G6PC* KO mice. **(a)** PCA aggregation analysis, which demonstrates the clustering of similar samples. Closer relative distances indicate lower variability between samples. **(b)** Heat map of differential gene cluster analysis. **(c)** Verification of upregulation trend in differential gene expression in WT and KO-6 groups. **(d)** Verification of downregulation trend in differential gene expression in WT and KO-6 groups. Significantly different values from WT group (*P < 0.05; **P < 0.01; ***P < 0.001) are indicated. Representative blots are shown.

To explore the mechanism underlying fatty liver formation in *G6PC* KO mice, we performed transcriptome sequencing using RNA samples from WT, KO-3, KO-6, and KO-9 mice. The PCA plot demonstrated that the WT group was more similar to the KO-3 group but separated from the KO-6 and KO-9 groups (Figure 3a). Based on differential gene cluster analysis, the heat map revealed that gene expression in the WT mice was close to that of the KO-3 group; while the transcriptome expression profiles in liver tissues from the KO-6 and KO-9 groups were relatively close (Figure 3b). Differential expression analysis was conducted using DESeq and are shown in Supplementary Table S3.

To verify the differentially expressed genes in the RNA-seq data, five upregulated genes and five downregulated genes were randomly selected for qRT-PCR verification. As shown in Figure 3c, the mRNA expression levels of *pyruvate kinase L/R (PKLR)*, *LDL-receptor-related protein 4 (LRP4)*, *glycerol-3-phosphate acyltransferase 3 (GPAT3)*, *AACS*, and *ELOVL6* were all increased. The mRNA expression levels of *autophagy-related 16 like 2 (ATG16L2*), *phosphoenolpyruvate carboxy kinase 2 (PCK2)*, *lipoprotein lipase (LPL)*, *ethylmalonic encephalopathy 1 (ETHE1)*, and *regulator of calcineurin 2 (RCAN2*) were all decreased (Figure 3d). Thus, the results obtained from both qRT-PCR and RNA-seq analyses were consistent with each other. Cluster analysis of the differentially expressed genes demonstrated that the expression levels in the KO-3 group were similar to those in the WT group. Furthermore, there were no significant variations observed in the expression profiles of the associated tissue in the KO-6 and KO-9 groups.

### 3.4 Autophagic activity was impaired in *G6PC* KO mouse liver

Subsequently, we conducted GO enrichment analysis of each group. Results showed that the KO-3 group was mainly enriched in extracellular region and response to external stimulus (Supplementary Figure S3). The KO-6 group was mainly enriched in cytoplasm, intracellular, protein binding, and regulation of catalytic activity (Supplementary Figure S4). The KO-9 group was primarily enriched in monocarboxylic acid metabolic process (Supplementary Figure S5). Furthermore, the KO-6 and KO-9 groups collectively showed enrichment in the cellular component (CC) category related to lipid droplets, the molecular function (MF) category associated with the activity of lipid transporters, and the biological process (BP) category involved in lipid metabolism (Supplementary Table S4). This combined enrichment suggests a higher content of lipids in the bodies of mice in the KO-6 and KO-9 groups.

**FIGURE 4:**
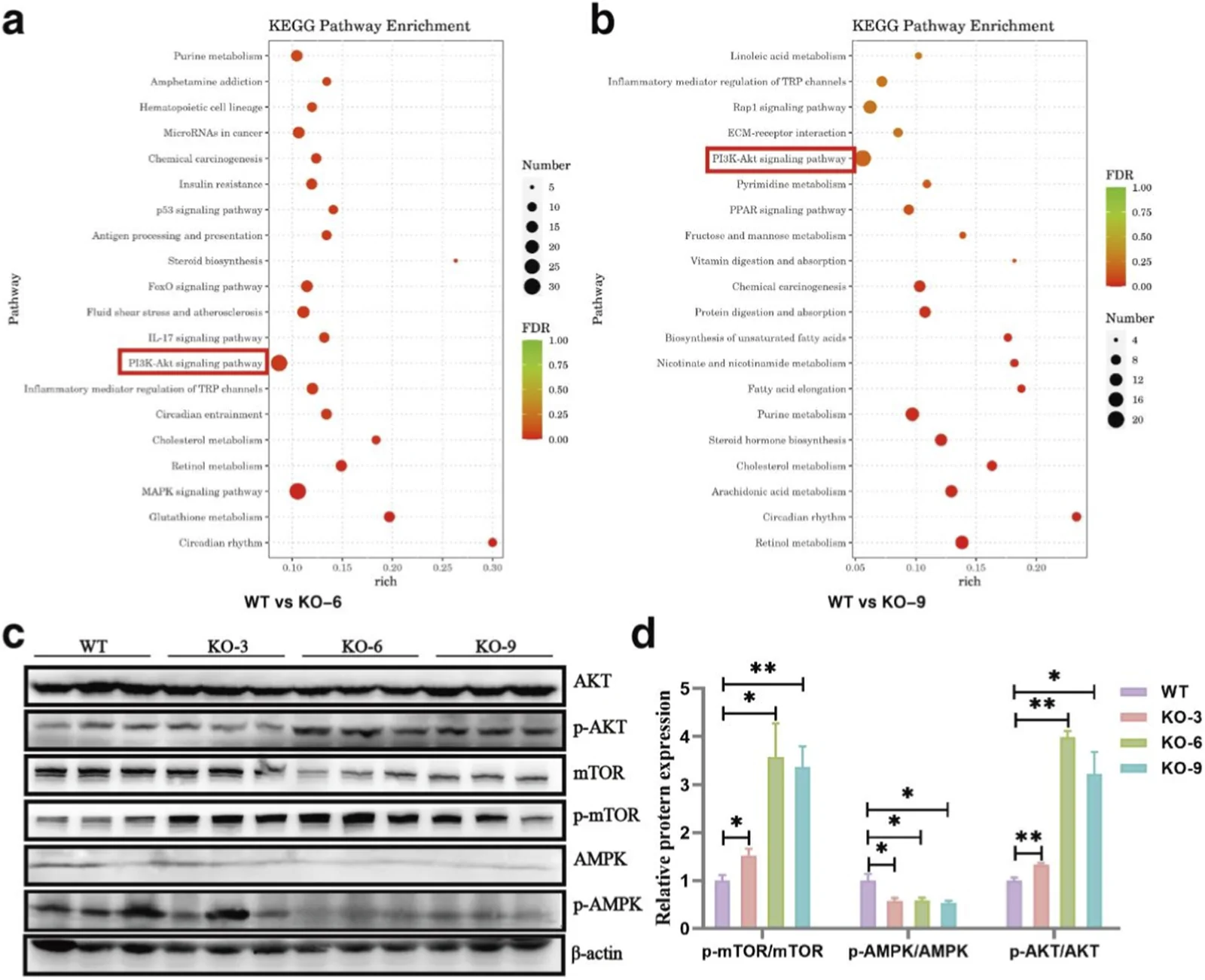
Signaling pathway analyses in *G6PC* KO adult mice. **(a)** Clustering analysis of KEGG pathways between *G6PC* KO 6-month-old mice and WT mice showing differential genes clustered in PI3K-Akt signaling pathway. **(b)** Clustering analysis of KEGG pathways between *G6PC* KO 9-month-old mice and WT mice showing differential genes clustered in PI3K-Akt signaling pathway (right). **(c)** Representative Western blot images of autophagy-related proteins and **(d)** relative protein expression levels (*P < 0.05; **P < 0.01; n = 6). Quantitative analysis was performed using independent biological replicates (n = 3) and normalized to β-actin.

Given that the KO-6 and KO-9 groups exhibited the most significant changes in gene expression and hepatic fatty phenotype, we focused our assessment of the altered signaling pathways in these groups. KEGG enrichment analysis demonstrated significant alterations in fat metabolism pathways in the KO-6 group. In the KO-9 group, KEGG pathway analysis revealed a substantial enrichment in the fat metabolism pathways, including steroid biosynthesis, linoleic acid metabolism, fatty acid elongation and so on. Additionally, KEGG pathway analysis revealed enrichment in the PI3K-Akt signaling pathway in both the KO-6 and KO-9 groups (Figures 4a,b).

Given the pronounced development of fatty liver in these groups, we postulated that PI3K-Akt signaling may affect fatty liver formation in *G6PC* KO mice. To explore this hypothesis further, we evaluated the level of phosphorylated AKT (p-AKT) in liver tissue. Results showed that p-Akt and insulin were significantly increased in the *G6PC* KO mice compared to WT mice, indicating activation of the Akt signaling pathway (Figures 4c,d). Moreover, our RNA-seq results suggested that autophagy-related pathways, such as AMPK, were involved in fatty liver development. Western blot analysis revealed that the phosphorylation levels of AMPK were noticeably reduced in the liver tissue of *G6PC* KO mice compared to those of the WT group. In contrast, the phosphorylation levels of mTOR and S6K1 were significantly increased in the *G6PC* KO group. These findings collectively indicate that autophagy activation was impaired in the livers of the *G6PC* KO mice.

### 3.5 PPAR-α agonist bezafibrate enhanced autophagic activity impaired by *G6PC* KO

**FIGURE 5:**
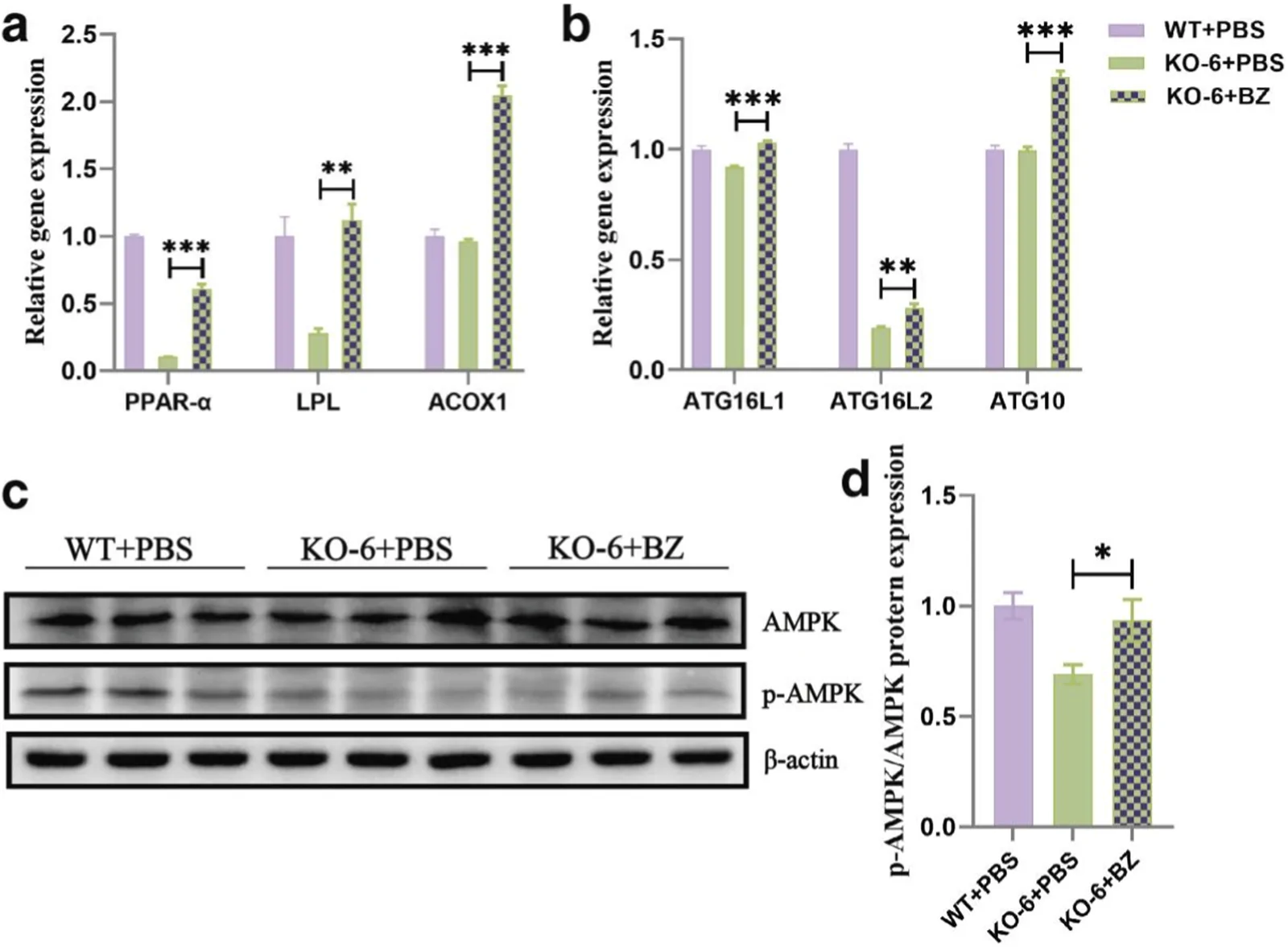
PPAR-α agonist bezafibrate enhanced autophagic activity impaired by *G6PC* KO. **(a)** mRNA expression levels of *PPAR-α* and its target genes, *LPL* and *ACOX1*.**P < 0.01; ***P < 0.001; n = 6. **(b)** Levels of autophagy-related genes *ATG16L1*, *ATG16L2*, and *ATG10*. **P < 0.01; ***P < 0.001; n = 6. **(c)** Protein expression levels of p-AMPK and AMPK in liver. **(d)** Relative protein expression levels of p-AMPK/AMPK in liver (*P < 0.05). Quantitative analysis was performed using independent biological replicates (n = 3) and normalized to β-actin.

Bezafibrate, a potent PPAR-α agonist, plays a key role in regulating lipid metabolism by modulating the expression levels of genes involved in fatty acid oxidation. To examine the effects of bezafibrate intervention, we assessed the mRNA levels of PPAR-α and its target genes, *LPL* and acyl-CoA oxidase 1 (*ACOX1*), after 1 month of treatment. Our results demonstrated that bezafibrate treatment rescued the decreased expression of PPAR-α caused by *G6PC* KO. Additionally, the mRNA expression levels of *LPL* and *ACOX1* were persistently elevated in the livers of the bezafibrate-treated mice compared with the *G6PC* KO mice (Figure 5a). Thus, these findings suggest that bezafibrate has the potential to rescue the expression levels of PPAR-α and its target genes.

Autophagy is a crucial cellular process involved in nutrient recycling and defense against pathogens and harmful stimuli in animals. Here, we subsequently investigated whether bezafibrate has an impact on the expression of autophagy-related genes (ATGs). As shown in Figure 5b, bezafibrate significantly increased the hepatic expression of these ATGs. Furthermore, we performed Western blot analysis to determine the expression levels of autophagy-related pathways. Results showed a significant decrease in the expression ratio of *p*-AMPK/AMPK in the *G6PC* KO group, but an increase in expression in the presence of bezafibrate (Figures 5c,d). Taken together, these findings suggest that bezafibrate may enhance autophagic activity impaired by *G6PC* KO.

### 3.6 PPAR-α agonist bezafibrate attenuated inflammatory response, fat deposition, and glycogen storage in global *G6PC* KO mice adult mice

**FIGURE 6:**
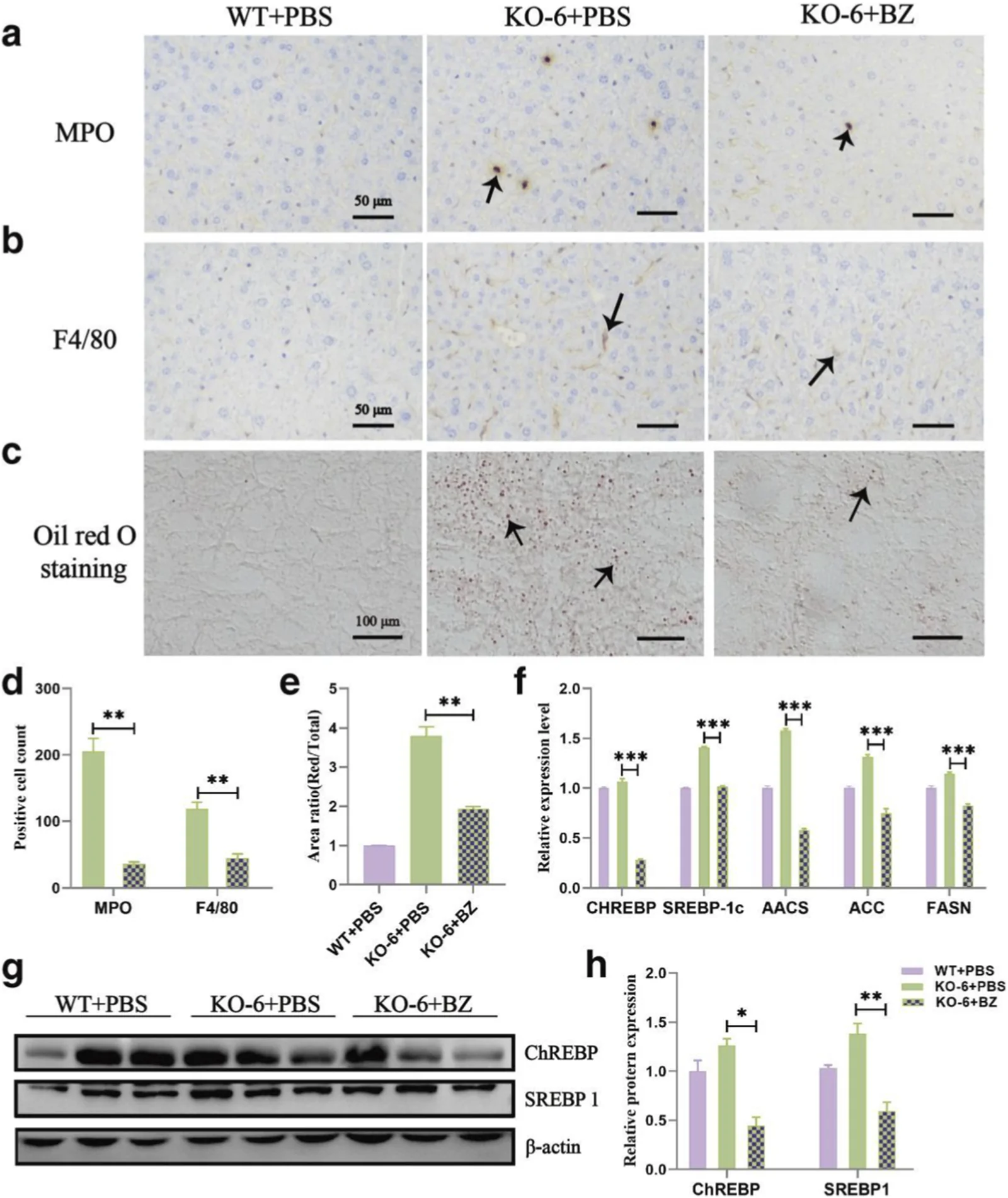
Effect of bezafibrate on inflammatory response, fat deposition, and glycogen storage in global *G6PC* KO adult mice. **(a,b)** Immunohistochemical staining of MPO and F4/80. Scale bar = 50 μm. **(c)** Oil Red O staining. Scale bar = 100 μm. **(d)** Quantification of positive cell number in a and b. **P < 0.01; n = 6. **(e)** Quantitative analysis of Oil Red O staining intensity. Percentages of brick red oil droplets in total area. **P < 0.01; n = 6. **(f)** Expression of adipogenesis-regulating genes. ***P < 0.001; n = 6. **(g)** Representative Western blot images of ChREBP and SREBP1 in liver and **(h)** relative expression levels of ChREBP and SREBP1 proteins. Quantitative analysis was performed using independent biological replicates (n = 3) and normalized to β-actin.

The number of MPO-positive cells in the bezafibrate-treated *G6PC* KO adult mice was lower than that in untreated mice, indicating that bezafibrate administration decreased the number of neutrophils in global *G6PC* KO adult mice (Figures 6a,d). Immunohistochemical analysis revealed a decrease in F4/80-positive cells in the livers of bezafibrate-treated *G6PC* KO adult mice compared to untreated *G6PC* KO adult mice, indicating a decrease in the number of macrophages in the liver (Figures 6b,d).

Furthermore, Oil Red O staining demonstrated a reduction in oil droplets in the livers of the bezafibrate-treated *G6PC* KO adult mice compared to the untreated *G6PC* KO adult mice (Figures 6c,e). To further explore whether bezafibrate repaired liver steatosis in *G6PC* KO adult mice, adipogenic regulatory factors in the liver of *G6PC* KO adult mice were assessed. The qRT-PCR results showed a decreasing trend in the expression levels of *ChREBP*, *SREBP1*, *AACS*, *ACC*, and *FAS* in the livers of the bezafibrate-treated *G6PC* KO adult mice (Figure 6f). Western blot analysis also showed a decreasing trend in the protein expression levels of ChREBP and SREBP1c in the livers of the bezafibrate-treated *G6PC* KO adult mice compared to untreated *G6PC* KO adult mice (Figures 6g,h). In summary, these findings indicate that treatment with bezafibrate can mitigate fat deposition in the livers of the *G6PC* KO adult mice.

## 4 Discussion

Maintaining normal blood sugar levels is crucial for preventing and controlling the adverse development of GSDIa. Since the 1970s, slow glucose release dietary therapy based on corn starch has enabled some GSDIa patients to survive into adulthood (Derks et al., 2021). In addition, gene therapy has shown effectiveness in several *G6PC*-deficient animal models (Arnaoutova et al., 2021; Kishnani et al., 2019; Zingone et al., 2000). However, obtaining a global *G6PC* KO adult animal without relying on gene therapy remains a challenge. In a previous study, Salganik et al. achieved a 60% survival rate of *G6PC* KO pups through weaning using glucose injections, glucose fortified water, and food supplementation (Salganik et al., 2009). However, glucose injections are time-consuming, labor-intensive, and prone to deviations in results if not performed properly. In the present study, we employed a “surrogate breast feeding” strategy to provide sufficient care and milk for *G6PC* KO pups and achieved a 24.2% (23/95) survival rate during the weaning process. The surviving pups successfully overcame the critical weaning period, showed compensatory growth in body weight, and exhibited relatively normal mobility and activity. We hypothesize that G6Pase-β, an isoenzyme of G6Pase-α, may contribute to the improved survival rate. Although G6Pase-β exhibits only 14% of the catalytic efficiency of G6Pase-α, it is widely expressed in both the liver and muscle tissue (Jaiswal et al., 2019; Wang et al., 2013).

Liver steatosis, characterized by the excessive accumulation of fat in the liver, is a prominent clinical feature of human GSDIa disease. Previous studies have reported a high prevalence of hypertriglyceridemia (98.6%) and hypercholesterolemia (58.3%) (Zhang et al., 2020), as well as liver fatty lesions (45.5%), in GSDIa patients (Göğüş et al., 2002). However, Salganik et al. found that adult global *G6PC* KO mice display liver histology disorder and glycogen accumulation, but no fatty liver formation (Salganik et al., 2009). In the present study, we demonstrated that global *G6PC* KO adult mice exhibited typical fatty liver phenotypes, including fat deposition in the liver, inflammatory cell invasion, and increased serum FFA, TG, and CHO levels. These phenotypes are consistent with the characteristics of fatty liver and indicate that our *G6PC* KO model can serve as a platform for studying fatty liver development in human GSDIa.

Deficiency in G6Pase-α in GSDIa causes the accumulation of G6P in the liver, which plays a key role in glucose and lipid metabolism and transformation. Firstly, G6P is transformed into pyruvate through the glycolysis pathway, which is then converted into acyl-CoA, a lipid synthesis substrate, through the tricarboxylic acid cycle in the mitochondria. Subsequently, acyl-CoA participates in fat synthesis by being transformed into FFA and TG with the involvement of various lipid regulatory enzymes (Moonira et al., 2020). Secondly, several studies have highlighted the ability of G6P to activate ChREBP, which regulates the downstream expression of various enzymes involved in fat synthesis (Hoogerland et al., 2019; Lei et al., 2020; Rajas et al., 2021). For example, Lei and Rajas et al. revealed that hepatic ChREBP is activated in *G6PC*-deficientmice, with its knockdown/KO found to protect against non-alcoholic fatty liver disease (Rajas et al., 2021). Consistent with these results, we observed G6P accumulation in the livers of global *G6PC* KO mice, which activated the transcriptional regulator ChREBP for fat synthesis, resulting in an increase in the expression levels of fat synthases. In addition, SREBP1, another important transcription factor involved in lipid synthesis and lipid metabolism gene regulation, was also increased in our global *G6PC* KO mouse model (Deng et al., 2014). Collectively, these studies suggest that *G6PC* deletion can lead to the activation of the *de novo* fat synthesis pathway, thereby promoting the formation of fatty liver in GSDIa mice.

The accumulation of fat in the liver is influenced not only by fat synthesis but also by fat degradation mediated by autophagic processes (Cho et al., 2021; Gautam et al., 2020; Waskowicz et al., 2019). The autophagy pathway is regulated by AMPK and mTOR, both of which are implicated in the development of GSDIa fatty liver. In our study, we observed decreased AMPK activity and increased mTOR activity, leading to impaired autophagy and reduced lipolysis. Similarly, Farah et al. also reported a decline in autophagic activity during the development of fatty liver in newborn *G6PC* KO mice, which was associated with the downregulation of AMPK pathway activity and upregulation of mTOR pathway activity (Farah et al., 2016). Gautam et al. demonstrated that upregulation of the AMPK pathway enhanced liver autophagic activity in *G6PC* KO mice, promoted fat breakdown, and slowed the development of GSDIa fatty liver (Gautam et al., 2020). Thus, our findings, along with previous studies, indicate the involvement of autophagy impairment in the formation of GSDIa fatty liver.

KEGG pathway analysis of the livers of KO-6 and KO-9 mice showed marked changes in the activity of various signaling pathways, such as PI3K-Akt, AMPK, and MAPK. Prior research has established that Akt signaling modulates cellular autophagic activity via two distinct pathways. The first pathway involves the well-documented Akt/mTOR/S6K1 axis, which is associated with impaired autophagy signals (Xu et al., 2020). The second pathway involves the attenuation of cellular autophagic activity by promoting the ubiquitin-mediated degradation of AMPK via the inhibition of AMPK phosphorylation at Ser485/491 (Suzuki et al., 2013). Consistent with our RNA-seq results, the expression level of p-Akt was significantly increased in the KO-6 and KO-9 mice.

PPAR-α controls the transcription of many genes involved in lipid catabolism. By binding to and activating PPAR-α, bezafibrate can induce lipogenesis and promote mitochondrial function (Seminotti et al., 2023). While several studies have suggested that bezafibrate can have therapeutic effects on *G6PC* KO mice surviving up to 2 weeks, the effects of bezafibrate on adult GSDIa mice have not been previously mentioned (Kang et al., 2019). Our study provided evidence that the PPAR-α agonist bezafibrate attenuated inflammatory responses, fat deposition, and glycogen storage in adult GSDIa mice. However, the inflammatory evaluation in this study was mainly limited to hepatic markers of inflammatory cell infiltration. Additional assessment of circulating cytokines, such as TNF-α and IL-6, would help to better define the systemic inflammatory status and should be included in future studies. The beneficial effects of bezafibrate observed in this study are biologically plausible given the known role of PPAR-α in promoting fatty acid oxidation and improving hepatic lipid handling. In addition, PPAR-α signaling has been reported to interact with autophagy-related pathways, suggesting that its activation may influence both lipid utilization and intracellular quality-control mechanisms (Reghelin et al., 2025; Waskowicz et al., 2019). Consistent with this possibility, bezafibrate treatment was associated with improvement of autophagy-related alterations in *G6PC* KO mice. However, because direct autophagic flux assays were not performed in the present study, the mechanistic link between PPAR-α activation and autophagy restoration requires further investigation. A limitation of the present study is that autophagy was evaluated mainly by marker expression rather than direct measurement of autophagic flux. Therefore, although bezafibrate treatment was associated with changes in autophagy-related proteins, the current data do not definitively establish restoration of autophagic flux. Future studies incorporating flux assays, such as lysosomal inhibition-based LC3 turnover analysis, will be needed. Another limitation of the present study is that only male mice were used. Given the known sexual dimorphism in lipid metabolism, future studies including both sexes are needed to evaluate the sex dependence of the observed hepatic phenotypes and treatment effects.

Moreover, a dedicated toxicity assessment of bezafibrate at the administered dose was not performed. The dose of 75 mg/kg used in this study was selected based on previously published *in vivo* mouse studies and preliminary efficacy considerations. In particular, prior work has reported that intraperitoneal administration of bezafibrate at 150 mg/kg once daily for 20 days was tolerated in mice, with no significant effect on body weight under those experimental conditions (Dumont et al., 2012; Wan et al., 2020). However, such published tolerability data cannot substitute for a systematic safety evaluation in our own model. Therefore, although the present findings support the biological efficacy of bezafibrate, future studies should include formal toxicity assessments, including longitudinal body weight monitoring, serum biochemical analysis, and histopathological examination of major organs, to better define its safety profile in this setting.

Although the surrogate-fed global *G6PC* KO mice used in this study exhibited a relatively low survival rate, this model has been reported to recapitulate key systemic and hepatic manifestations of GSD-Ia. We do not claim that this model is superior to liver-specific *G6PC* KO models, which are particularly useful for dissecting liver-intrinsic mechanisms with improved survivability. Rather, the present model was selected because it captures the severe metabolic context of GSD-Ia and was suitable for evaluating the hepatic pathological changes and treatment effects examined here. Nevertheless, because no direct comparison with liver-specific knockout models was performed, the relative advantages and limitations of each model warrant further investigation.

Importantly, caution is required when extrapolating these findings from mice to patients with GSDIa. Although the surrogate-fed global *G6PC* KO model reproduces several key metabolic and hepatic features of the disease, human GSDIa is a chronic and clinically heterogeneous disorder with variable metabolic control, long-term complications, and complex treatment requirements that are not fully captured in murine models. In addition, species differences in hepatic metabolism, lipid handling, and pharmacologic responsiveness may influence both the magnitude and mechanism of bezafibrate’s effects. Therefore, the therapeutic benefit observed in mice should be interpreted as preclinical evidence rather than a direct predictor of clinical efficacy in humans. The potential clinical application of bezafibrate also warrants careful consideration. Bezafibrate is an established lipid-lowering agent with prior clinical use in humans, which supports the feasibility of drug repurposing. However, its use in GSDIa remains investigational, and direct clinical evidence in this patient population is currently limited. Although previous clinical studies have demonstrated the general tolerability and metabolic effects of bezafibrate in other settings, these data do not establish efficacy or safety in GSDIa, particularly given the unique metabolic features of this disorder and the need for long-term treatment. Future translational studies and, ultimately, dedicated clinical trials will therefore be required to determine the optimal dosing, safety profile, and therapeutic value of bezafibrate in patients with GSDIa.

In conclusion, we successfully employed surrogate breastfeeding to enable global *G6PC* KO mice to survive into adulthood. These mice exhibited typical fatty liver phenotypes, such as increased fat deposition, inflammatory response, and blood lipid levels, accompanied by dysregulation of transcriptional regulators involved in lipogenesis. We also identified the involvement of autophagy impairment regulated by the PPAR-α pathway in GSDIa fatty liver. Our study provides a valuable platform for investigating the mechanisms underlying GSDIa fatty liver formation, as well as insights and potential directions for its treatment.

## Data Availability

The original contributions presented in the study are publicly available. This data can be found here: European Nucleotide Archive (ENA), https://www.ebi.ac.uk/ena/browser/view/PRJEB124169 accession number PRJEB124169.
