# Supplementary material for: Generation of a glycogen storage disease mouse model with fatty liver and its treatment by PPAR-α agonist bezafibrate
Source: Front Pharmacol. 2026 Aug 31;17:1898810. doi: 10.3389/fphar.2026.1898810 (PMC13572639; doi:10.3389/fphar.2026.1898810)
Supplement: Supplementary file 1 [file Supplementaryfile1.docx]

**Table S1** Primer pairs for RT-qPCR analysis

| Gene | Forward primer 5’-3’ | Reverse primer 5’-3’ | Tm(℃) |
| --- | --- | --- | --- |
| *ACC* | gagaggggtcaagtccttcc | acatccacttccacacacga | 56 |
| *FASN* | accaccagagaccgttatgc | tgggttctagccagcagagt | 56 |
| *AACS* | agtgctggaggagaaggaca | agtgctggaggagaaggaca | 56 |
| *ELOVL6* | atgaacaagcgagccaagtt | cccagaatttgctgacaggt | 56 |
| *LRP4* | cccctctccagaaacctacc | aagcccgtgtccaataacag | 56 |
| *GPAT3* | gccattgtggaggatgaagt | ggaagcaatagcgcactagg | 56 |
| *PKLR* | agagtccatcgccaacattc | cacaatttccacctccgact | 56 |
| *ATG16L2* | atcagcgagatcccaaacac | ccttcaccacatccacacag | 56 |
| *PCK2* | ttggagagaatgctcgtgtg | tggagaacagctgactggtg | 56 |
| *LPL* | agggctctgcctgagttgta | ccatcctcagtcccagaaaa | 56 |
| *ETHE1* | acttccaacaaggctgtgct | cctcacagctgagggtaagc | 56 |
| *RCAN2* | ggactgttccggacctatga | agggggtgagatgaggaact | 56 |
| *PPAR-α* | aatgcaattcgctttggaag | ggccttgaccttgttcatgt | 56 |
| *ACOX1* | ttatgcgcagacagagatgg | ggcatgtaacccgtagcact | 55 |
| *ATG16L1* | caaatgcagcagaaggacaa | cagggcgtcatactcatcct | 56 |
| *ATG10* | ttcacagcagataggcgatg | tgcaggtctcgtcacttcag | 56 |
| *CHREBP* | acctgtctccccctcaaact | tgtcttctgaagcgtggttg | 56 |
| *SREBP-1c* | cactcagcagccaccatcta | gctgtcagcagcagtgagtc | 59 |
| *β-ACTIN* | agccatgtacgtagccatcc | ctctcagctgtggtggtgaa | 56 |

**Table S2 Off-target analysis of G6PC^−/−^ mice**

| Target sequence | Potential off-target site | Potential off-target sequence | Mutation |
| --- | --- | --- | --- |
| AGAGATGAATGGA | chr8-b：115895513 | AGtGATGcATGGAgTTACGAAGG | No |
| CTTACGATGG | chr4：5411192 | AGAGATGtATGGgCTTAaGAGGG | No |
|  | chr5：20049287 | AGAcATGAATGGACTTgCaAAGG | No |
|  | chr7：50500114 | AGAGATGAATGGtgTTAtGAGGG | No |
|  | chr8-a：34119796 | AGAGAaGAtTGGACTTAgGATGG | No |
| CAAGGTGGAAGA | chr2 ：173940890 | CAcGGTGGctGAACCCCTCTTGG | No |
| ACCCCTCTGGG | chr16：43838082 | CAAGGTGGAAGAAatCaTCTAGG | No |
|  | chr3：101113026 | CAAGGTGGAtGAACCCaaCTGGG | No |
|  | chr1：120183606 | CAAGGTGGAAagACCCCTtTTGG | No |
|  | chr17：38576524 | CAAGGTGtAAGAcCCCCTtTGGG | No |

**Figure S1** Sanger sequencing results of the PCR products of potential off-target sites in sgRNA1. (A) Agarose electrophoresis results after PCR amplification (M: DL 1000 Marker; a: WT mice; b: *G6PC*^−/−^ mice); (B) Upstream and downstream primer amplification sequences (yellow background for primer sequences; bold font for potential miss sequences).

**Figure S2** Sanger sequencing results of the PCR products of potential off-target sites in sgRNA2. (A) Agarose electrophoresis results after PCR amplification (M: DL 1000 Marker; a: WT mice; b: *G6PC*^−/−^ mice); (B) Upstream and downstream primer amplification sequences (yellow background for primer sequences; bold font for potential miss sequences).

**Table S3** Statistics of differentially expressed genes

| Group | Up-regulated | Down-regulated | Total |
| --- | --- | --- | --- |
| WT vs KO-3 | 235 | 140 | 375 |
| WT vs KO-6 | 537 | 458 | 995 |
| WT vs KO-9 | 368 | 278 | 646 |

**Figure S3** Term for GO enrichment analysis in *G6PC^−/−^* 3-month-old mice.

**Figure S4** Term for GO enrichment analysis in *G6PC^−/−^* 6-month-old mice.

**Figure S5** Term for GO enrichment analysis in *G6PC^−/−^* 9-month-old mice.

**Table S4** Some GO terms of KO-6and KO-9 be clustered together

| GO.ID | Term | Category |
| --- | --- | --- |
| GO:0005811 | lipid droplet | CC |
| GO:0032994 | very-low-density lipoprotein particle | CC |
| GO:0034385 | triglyceride-rich plasma lipoprotein particle | CC |
| GO:0034358 | plasma lipoprotein particle | CC |
| GO:0034364 | high-density lipoprotein particle | CC |
| GO:0008289 | lipid binding | MF |
| GO:0005319 | lipid transporter activity | MF |
| GO:0120013 | lipid transfer activity | MF |
| GO:0030169 | low-density lipoprotein particle binding | MF |
| GO:0140303 | intramembrane lipid transporter activity | MF |
| GO:0006629 | lipid metabolic process | BP |
| GO:0045017 | glycerolipid biosynthetic process | BP |
| GO:0046486 | glycerolipid metabolic process | BP |
| GO:0046890 | regulation of lipid biosynthetic process | BP |
| GO:0046889 | positive regulation of lipid biosynthetic process | BP |
